# Case Report: Targeting the Achilles’ heel of monomorphic epitheliotropic intestinal T-cell lymphoma

**DOI:** 10.3389/fonc.2026.1710764

**Published:** 2026-02-06

**Authors:** Song Xue, Hong-fei Gu, Fan Yang, Wei Zhang, Xi-yang Liu, Hui-peng Sun, Xing-yu Cao

**Affiliations:** 1 Lu Daopei Hospital, Beijing, China; 2 Hexiaoduo Beijing Health Management Co., Ltd, Beijing, China; 3 Tongji University School of Medicine, Shanghai, China; 4 Department of Hematology, Tongji Hospital, Tongji University School of Medicine, Shanghai, China; 5 Department of Bone Marrow Transplant, Hebei Yanda Lu Daopei Hospital, Langfang, China

**Keywords:** allogeneic hematopoietic stem cell transplantation, BCL2, EATL-2, monomorphic epitheliotropic intestinal T-cell lymphoma, venetoclax

## Abstract

**Background:**

Monomorphic epitheliotropic intestinal T-cell lymphoma (MEITL) is a rare and highly aggressive lymphoma with a dismal prognosis and no established standard therapy. Its frequent expression of BCL-2 provides a rationale for targeting this anti-apoptotic protein.

**Methods:**

We report the case of a 34-year-old female with refractory MEITL who failed prior lines of therapy, including CHOPE and gemcitabine/oxaliplatin (GemOx) combined with golidocitinib. Based on positive BCL-2 expression by immunohistochemistry, a salvage regimen combining venetoclax with Gemox was administered.

**Results:**

The treatment induced a rapid and significant clinical improvement. A follow-up PET/CT scan confirmed complete metabolic remission. The main adverse event was grade 4 neutropenia and thrombocytopenia with febrile neutropenia, attributable primarily to the Gemox backbone, which resolved with supportive care. The off-label use was approved by the institutional committee, and informed consent was obtained.

**Conclusion:**

To our knowledge, this is the first report of successful treatment of refractory MEITL with a venetoclax-containing regimen. This case validates BCL-2 as a actionable therapeutic target in MEITL. Future efforts should focus on optimizing combination partners for venetoclax to improve efficacy and tolerability. The rationale for exploring venetoclax as post-transplant maintenance therapy in MEITL is also discussed.

## Introduction

Monomorphic epitheliotropic intestinal T-cell lymphoma (MEITL) is a rare, aggressive primary intestinal T-cell lymphoma, previously classified as type II enteropathy-associated T-cell lymphoma (EATL). It constitutes less than 1% of all non-Hodgkin lymphomas (NHL) and primarily occurs in elderly males, with a median onset age of about 60 years. The clinical course is highly aggressive, leading to a poor prognosis with a median survival of only around 7 months (1). No established treatment regimen exists for MEITL. While chemotherapy protocols such as CHOP are employed, their efficacy is limited. For patients with MEITL, whose prognosis is extremely poor, expert consensus supports considering allogeneic stem cell transplantation (allo-HSCT) in the first remission, even in the absence of strong evidence (2). For patients with relapsed/refractory MEITL, participation in clinical trials is highly advised butoften difficult to achieve. A critical barrier is that pivotal trials for new drugs approved in relapsed/refractory peripheral T-cell lymphoma (PTCL) have seldom enrolled patients with the MEITL subtype. This lack of dedicated data translates to off-label use in practice, which yields variable and largely suboptimal outcomes (3). Given this unmet need, managing relapsed or refractory disease is a key research priority (3, 4). Notably, MEITL often demonstrates BCL-2 expression, rendering it a potential target for BCL-2 inhibitors (5, 6). Therefore, we present a case of refractory MEITL successfully treated with a regimen containing venetoclax.

## Case presentation

In September 2024, a 34-year-old female presented with unexplained upper abdominal pain and anorexia. Initial management at a local hospital with oral medication provided suboptimal relief. Her symptoms progressed to abdominal distension and colic. An abdominal CT scan revealed marked thickening of several small bowel segments with mild heterogeneous enhancement. Following this, a small bowel endoscopy with biopsy on October 15, 2024, led to a diagnosis of intestinal T-cell lymphoma. Staging workup, including bone marrow biopsy and whole-body PET/CT, classified the disease as Lugano stage II.

**Figure 1:**
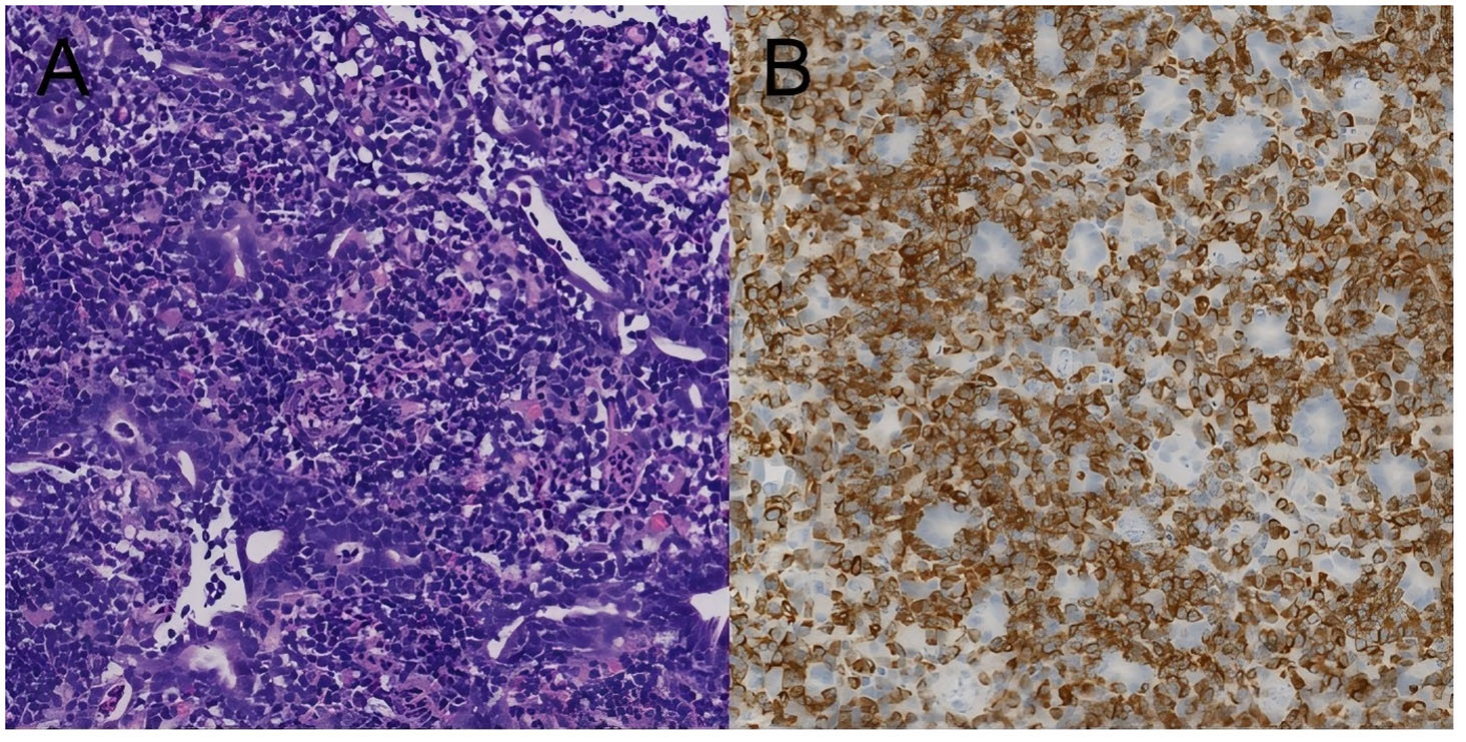
The pathological manifestations of the patient. **(A)** HE×200.**(B)** Bcl-2×200.

The patient subsequently received two cycles of CHOPE chemotherapy with minimal response, experiencing progressive intestinal obstruction. A subsequent cycle of the Gemox regimen combined with golidocitinib also proved ineffective. Due to persistent and severe abdominal symptoms, she was referred to our hospital for further management.Pathological review of the jejunal biopsy confirmed extensive infiltration by monomorphic, medium-sized atypical lymphocytes exhibiting epitheliotropism. The tumor cells had scant cytoplasm and round to slightly irregular nuclei with inconspicuous nucleoli (**Figure 1**). Immunohistochemistry was positive for CD3, CD2 (partial), CD7, CD43, CD56, CD8, TIA-1, and BCL-2, with a high Ki-67 index (>50%). The staining was negative for CD4, CD5, CD10, BCL-6, CD20, PAX5, CD79a, Cyclin D1, CD30, CD38, Granzyme B, and EBER. A diagnosis of MEITL was made.

**Figure 2:**
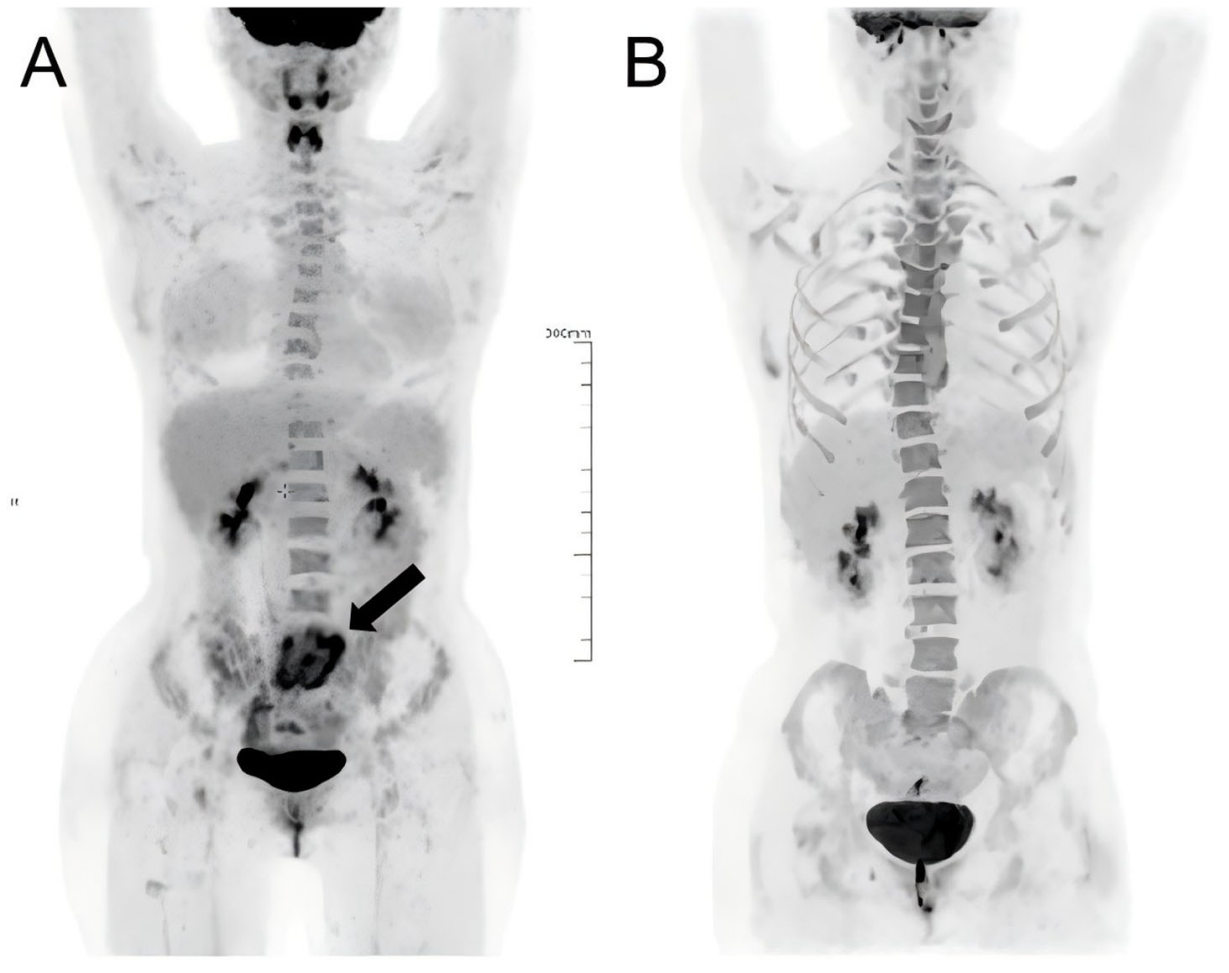
Comparison of PET/CT scans conducted **(A)** pre- and **(B)** post-treatment showed a high-uptake lesion in the patient’s abdomen (marked by the black arrow) prior to treatment, which resolved following treatment.

Given the tumor’s BCL-2 expression, a BCL-2-targeted combination regimen was initiated, consisting of venetoclax (400 mg, days 1-7), gemcitabine (1000 mg/m², day 7), and oxaliplatin (130 mg/m², day 7). Following treatment, the patient’s abdominal pain and distension significantly improved. However, she developed grade 4 neutropenia and thrombocytopenia accompanied by febrile neutropenia, which resolved with aggressive anti-infective therapy. After recovery, a second identical cycle was administered. A follow-up PET/CT scan demonstrated complete metabolic remission (**Figure 2**). The patient is currently undergoing allo-HSCT. The off-label utilization of venetoclax has been authorized by the Pharmaceutical Affairs Committee of Beijing Lu Daopei Hospital. The patient provid written informed consent for publication of this report and accompanying images.

## Discussion

To our knowledge, this is the first report describing the successful use of venetoclax in MEITL. This advance addresses a critical unmet need. The prognosis of untreated MEITL is dismal, with most patients succumbing within a month of diagnosis (4), despite rare documented cases of survival without systemic therapy (7). Although CHOP-like regimens remain the most common initial therapy, a large European retrospective study revealed poor outcomes, with an 86% disease progression rate and near-uniform mortality after progression (4). Salvage options are limited: CD30-targeted therapies like CAR-T, effective in some EATL cases (8), are seldom applicable due to infrequent CD30 expression in MEITL, and other salvage regimens yield negligible response rates (3).

The rationale for BCL-2 inhibition in MEITL is supported by its frequent protein expression, as reported in several smaller studies (5, 6, 9), even though large-scale retrospective analyses have not systematically examined this marker (4, 10). Notably, BCL-2 overexpression in MEITL is typically driven by transcriptional dysregulation and epigenetic mechanisms secondary to highly prevalent SETD2 loss-of-function mutations-not by the chromosomal t(14;18) translocation (2, 4, 11). This suggests immunohistochemistry (IHC) may be a sufficient and reliable method for detecting this target, potentially obviating the need for routine next-generation sequencing (NGS) testing in this context.

This case demonstrates that targeting BCL-2 with venetoclax can induce a profound response in refractory MEITL. The severe cytopenia observed was likely attributable mainly to the gemcitabine and oxaliplatin backbone of the combination. Consequently, future research should focus on identifying optimal chemotherapeutic or non-chemotherapeutic partners for venetoclax to enhance efficacy while more effectively managing treatment-related toxicity.This underscores the importance of conducting rigorously designed clinical trials to investigate safer dosages and further confirm the safety and effectiveness of the treatment.

The experience with allogeneic hematopoietic stem cell transplantation in MEITL is still limited. However, the reports of disease relapse following transplantation are a significant concern (12). This underscores the difficulty in treating MEITL and suggests a necessity for maintenance strategies after allo-HSCT. The potential utility of venetoclax in MEITL, combined with its proven role in reducing relapse as post-allo-HSCT maintenance for high-risk AML (13), provides a strong rationale. Therefore, adopting a venetoclax maintenance regimen for MEITL patients after transplant, analogous to the practice in high-risk AML, is a viable clinical consideration.

## Data Availability

The original contributions presented in the study are included in the article/supplementary material. Further inquiries can be directed to the corresponding author.
